# Again a Sale of American Diplomas in Europe

**Published:** 1885-03

**Authors:** 


### Again a Sale of American Diplomas in Europe.

The following editorial article appears in the edition of the *Deutzche Medizinal Zeitung* (one of the best, if at present not the best German medical journal,) of December 22, 1884. As it can only be of advantage to the medical and dental profession of our country to make the swindle as widely known as possible, and to denounce it in public journals, we bring in the following, a verbatim translation of the article in question, whereby we cannot conceal that affairs of this kind by no means contribute to our reputation in Europe.

" *Swindle—Doctor—Diplomas.* The *German Monthly Journal for Dentistry*, 1884, publishes the following interesting communication of Dr. Adolph Petermann, in Frankfort-on-Main:

The *Frankfort Daily* brought in its advertising columns the following announcement:

" Doctor-diplomas of Dentistry, Philosophy, Jurisprudence, etc., are reliably and discreetly procured. Address, C. R. (care of Stationer), 10 Duke street, Bloomsbury, London, W. C."

An inquiry under a fictitious name was responded to from London by two letters from a "Professor" G. Rumler. The first letter is not worth publishing, containing but a general offer and assuring discretion. The second letter was as follows:

LONDON, 32 THORNHILL CRESCENT,<br>BARNSBURY, March 10, 1884.

*Dear Sir:* In answer to your favor, I respectfully inform you that you have to deal with an honest man, who will procure you the diploma only from a reliable institute, legally authorized to issue diplomas. The name of the institute is: "Wisconsin Dental Academy."

You may deposit the fee with any banking firm in Frankfort, and have the deposit certified to by the firm and communicated to me, whereupon I shall at once order the diploma for you. If you do not wish to confide in a banking house of your city, it will be best if you will send to me 200 marks (at about $70) once, and the remaining $70 on receipt of the diploma. Any other expenses besides the $140 you have not. Please inform me, when ordering the document, of all your Christian names.

Further, I beg you to sign the enclosed printed application for graduation at the place where you read the word, "signed." All the rest I shall fill out. You will see at the back of the blank the attest of the President of the Academy that the degree is only conferred, if recommended *by me*. It will take about five weeks ere I can deliver the diploma to you.

Awaiting your reply and the return of the application signed, I am, etc., etc.,

PROF. DR. G. RUMMLER.

The words on the back were *written* as follows:

"DELAVAN, WIS., U. S. A.

"This statement will be honored from any practicing dentist if endorsed by Prof. Dr. Rummler.

GEO. MORRISON, *President*."

The "honest man," "Professor Dr." Rummler, therefore procures anybody (also under a fictitious name), without trouble, for a payment of $140, and *without any scientific attainments or examinations*, a doctor-diploma from a chartered Wisconsin Dental College! Nice state of affairs!

From a former article of mine on the same subject, it will be seen that Mr. Morrison delivers even for $12 such a diploma, while here $140 are demanded. Indeed Mr. Rummler is engaged in a dirty, but profitable business.

Amongst the advertisements of the *Kladderadatsch* is the following:

"Doctor Diplomas of Dentistry. Their legality officially attested. Discrete and reliable. B. Walden, 41 Princes Square, Kansington Park, London, S. E."

On inquiry at the above address, one receives the reply from Berlin from Dr. Olschowsky, 100 Linden street. Mr. O. is gladly willing to procure the doctor-diploma from an American university, respectively a dental college, whose sole representative he is. After he has mentioned that the institute is legally entitled to confer "honorary doctor titles," he continues, verbatim, as follows: " You are permitted, therefore, to call yourself a doctor here, if you only add to it: 'Conferred in a foreign country,' or, 'not conferred here.' You may see in every Berlin newspaper, especially the *Tageblatt*, that they daily contain many advertisements of this kind, with these words added: "To procure you the diploma I need for the beginning only a short curriculum of your life, and I shall then take care of the rest and send you the diploma all filled out, so that you need neither interrupt your daily occupation, nor do anything whatever."

Finally, O. informs the applicant that the whole expenses amount to $200, and that pre-payment is not needed, only the deposit of the sum with a reliable business firm, but best with W. Marzillier & Co., Berlin, S. W., 25 Morkern str.

On further inquiry, Dr. Olschowsky answers, that all these things "must pass through his hands, as the college does never correspond directly with anybody else," and "that the college in question was situated in Delavan." In a third letter the application for promotion arrived, in a fourth Dr. O. advises quickly to conclude the affair, and in the fifth letter Dr. O. tries to quiet any fears of the questioner, in so far as he tells him that Prof. Morrison, the President of the Wisconsin Dental College, comprehends by practicing dentist only dental technic, and that dental surgeon meant something else. Dr. O. concludes verbatim. " It says, therefore, in the diplomas, which I shall obtain for you, not Doctor of Dentistry, but Doctor of Dental Surgery. Besides I have already procured this diploma for applicants, of whom the gentlemen across the ocean knew that they were not dentists, and it is therefore the same with you. I hope to have removed your scruples, and expect immediate conclusion of the affair, which opportunity I use to inform you that $100 will meanwhile suffice."

Dr. Olschowsky, who has already formerly been active in the distribution of Buchanan's (the so-called Philadelphia) diplomas, now endeavors, therefore, to sell the totally valueless honorary doctor-diploma of the *Wisconsin Dental College, in Delavan, Wis-*

*consin, U. S. A.*, at the price of $200, while the same bogus diplomas are procured through Prof. Dr. Rummler, in London, for $140, and from the Wisconsin Dental College itself for $12. I have sent the letters of Dr. Olschowsky to the Imperial Secretary of the Interior (of Germany), and thus we may hope that from there this dirty trade may be suppressed.

This barter and sale of American diplomas in Europe ought to be thoroughly exterminated at its source, *i. e.*, in our country; and it would be desirable that all legislatures, or Congress, should pass the the most stringent laws, not only declaring the charter of such swindling institutions null and void, but also punishing with penitentiary at solitary confinement and hard labor, for a long period of years, any violation of the law. Of late years our profession has striven most earnestly and successfully to occupy the same rank with its European confreres. American dentistry is recognized all over the world as far ahead of that of other countries; but if American physicians and dentists permit the existence of such swindling institutions, and the sale of bogus diplomas to continue under their very noses, then they must not feel offended if they, on visiting Europe, are looked upon with suspicion, if no special credentials convince their professional brethren across the water that they (the American physicians and dentists) really are graduates of a respectable college.

It is scarcely possible that there can be many such diploma manufactories, and the few that still vegetate ought to be easily eradicted.

---

EPISTAXIS—HOW TO CHECK IT.—Dr. Henry Perdue, of Barnesville, Ga., plugs the bleeding nose with pieces of fat bacon applied the whole length of the nares. He secures the plugs by piece of string, and, if necessary, prevents them from falling out by a strip of adhesive plaster over the orifices of the nose. He gives particulars of three cases in which this method of treatment proved most successful when the ordinary plan of plugging and other methods had proved useless. These cases are only samples of others. He has yet to record a failure.—*Med. Press.*

